# Chromophores’ Contribution to Color Changes of Thermally Modified Tropical Wood Species

**DOI:** 10.3390/polym15194000

**Published:** 2023-10-05

**Authors:** Tereza Jurczyková, Ondřej Šárovec, František Kačík, Kateřina Hájková, Tomáš Jurczyk, Richard Hrčka

**Affiliations:** 1 Department of Wood Processing and Biomaterials, Faculty of Forestry and Wood Sciences, Czech University of Life Science Prague, Kamýcká 129, 165 21 Prague, Czech Republic; ondrejsarovec@seznam.cz (O.Š.); hajkovakaterina@fld.czu.cz (K.H.); 2 Department of Chemistry and Chemical Technology, Faculty of Wood Sciences and Technology, Technical University in Zvolen, T. G. Masaryka 24, 960 01 Zvolen, Slovakia; kacik@tuzvo.sk; 3 TIBCO Software s.r.o., Klimentská 1216/46, 110 00 Prague, Czech Republic; tjurczyk@tibco.com; 4 Department of Wood Science, Faculty of Wood Sciences and Technology, Technical University in Zvolen, T. G. Masaryka 24, 960 01 Zvolen, Slovakia; hrcka@tuzvo.sk

**Keywords:** ThermoWood, chemical degradation, extractives, optical properties, UV-Vis diffuse reflectance, total color difference

## Abstract

This work examines the effect of thermal modification temperature (180, 200, and 220 °C) in comparison with reference (untreated) samples on selected optical properties of six tropical wood species—Sp. cedar (*Cedrala odorata*), iroko (*Chlorophora excelsa*), merbau (*Intsia* spp.), meranti (*Shorea* spp.), padouk (*Pterocarpus soyauxii*), and teak (*Tectona grandis*). The main goal is to expand the existing knowledge in the field of wood thermal modification by understanding the related degradation mechanisms associated with the formation of chromophoric structures and, above all, to focus on the change in the content of extractive substances. For solid wood, the CIELAB color space parameters (L*, a*, b*, and ΔE*), yellowness (Y), ISO brightness, and UV-Vis diffuse reflectance spectra were obtained. Subsequently, these wood samples were extracted into three individual solvents (acetone, ethanol, and ethanol-toluene). The yields of the extracted compounds, their absorption spectra, and again L*, a*, b*, ΔE*, and Yi parameters were determined. With increasing temperatures, the samples lose brightness and darken, while their total color difference grows (except merbau). The highest yield of extractives (mainly phenolic compounds, glycosides, and dyes) from thermally modified samples was usually obtained using ethanol. New types of extractives (e.g., 2-furaldehyde, lactones, formic acid, some monomer derivatives of phenols, etc.) are already created around a temperature of 180 °C and may undergo condensation reactions at higher temperatures. For padouk, merbau, teak, and partially iroko modified at temperatures of 200 and 220 °C, there was a detected similarity in the intensities of their UV-Vis DR spectra at the wavelength regions corresponding to phenolic aldehydes, unsaturated ketones, quinones, stilbenes, and other conjugated carbonyl structures. Overall, a statistical assessment using PCA sorted the samples into five clusters. Cluster 3 consists of almost all samples modified at 200 and 220 °C, and in the other four, the reference and thermally modified samples at 180 °C were distributed. The yellowness of wood (Y) has a very high dependence (r = 0.972) on its brightness (L*) and the yellowness index of the extractives in acetone Yi(Ac), whose relationship was described by the equation Y = −0.0951 × Y(Ac) + 23.3485.

## 1. Introduction

Wood has multipurpose uses that are impossible to deny and it is difficult to note all of them in our daily lives. Wood is used commercially worldwide for construction and fencing, furniture, floors, household uses, shipbuilding, artworks, musical instruments, wooden toys, etc. Among the characteristics that are evaluated first is its color—a visual perception influenced by the spectral composition of the reflected light rays. The incident rays are partially absorbed by the wood chromophores, i.e., parts of the molecules capable of integrating with ultraviolet (UV) and visible (Vis) wavelengths of electromagnetic radiation [1]. As a result, chromophoric structures are responsible for the color of wood, being part of all its main components (lignin as well as polysaccharide parts) and extractive substances, more significantly represented in tropical wood species [2]. Their usually low concentration is increased by the depolymerization, condensation, or oxidation of individual wood components due to stressful situations [3,4,5].

One such situation is the exposure of wood to heat, which uses thermal modifications with the aim of improving its selected properties compared to unmodified wood and increasing its application. This process usually takes place in a thermal chamber in an environment of air, steam, nitrogen gas, hot oil, or vacuum, most often at a temperature between 160 and 240 °C. The structural arrangement of thermally treated wood occurs firstly at its molecular chemical level, mainly due to the degradation of hemicelluloses, the cleavage of hydroxyl groups from hemicelluloses and lignin, and the 3D spatial crosslinking of hemicelluloses with highly thermally stable lignin. At the same time, the essence of thermal modification is targeted intervention in its chemical composition and structure, which also affects the color and representation of chromophoric structures [6,7,8,9]. The discoloration reactions that occur during heat treatment are often explained by the formation of colored oxidation and degradation products concerning the main wood components and extractives. In lignin, there is a cleavage of β-O-4 linkages, resulting in a higher concentration of phenolic groups and a demethoxylation, leading to a more condensed structure via lignin autocondensation. Changes in phenolic extractives have been identified as another probable reason for the discoloration of wood. Temperature and oxygen cause polymerization of ellagitannins, while vacuum drying effectively prevents discoloration due to a lack of oxygen. The first part of the color change process is because of the formation of chromophoric groups such as carbonyl and carboxyl groups resulting mainly from the degradation of carbonyl, biphenyl, and ring-conjugated double bond structures in lignin. The release of acids and formation of quinoid compounds and carboxylic groups during heat treatment were also confirmed [10,11,12,13].

Thermally modified wood is not just an academic topic these days. Thermal wood treatment has already been penetrating the market for several years. The most common heat-modified woods in North America and Europe include red oak, Norway spruce, European and North American white ash, hemlock, radiata and Scots pine, beech, poplar, etc. The typical dark tones of these heat-treated wood species increase their economic value [14,15,16,17,18]. There is still some input into research and development. For example, the thermal modification of tropical wood species is a relatively young topic, but it is the subject of interest for many researchers: Kačíková et al. [19,20] dealt with chemical changes in lignin due to thermal modification in meranti, padouk, merbau, teak, and iroko wood. Gašparík et al. [21] and Gaff et al. [22] evaluated the impact of thermal modification on color, mechanical, and chemical changes in teak, meranti, padouk, merbau, iroko, and mahogany wood. Korkut [23] assessed the performance of three thermally treated tropical woods (sapele, limba, and iroko) commonly used in Turkey based on changes in density, weight loss, swelling, color difference, surface, and physical properties. Mburu et al. [24] characterized the physicochemical properties of heat-treated Southern silky oak (*Grevillea robusta*), a tropical wood species with a low natural durability, to improve its decay resistance. Lengowski et al. [25] compared the anatomical, chemical, physical, mechanical, colorimetric, and thermal stability properties of thermally treated teak (*Tectona grandis*) with the characteristics of the untreated teakwood to understand the effects of the thermal modification technique beyond color changes. Esteves et al. [26] investigated the changes in the content and composition of lignin, cellulose, hemicelluloses, and extractives in dichloromethane, ethanol, and water in thermally modified African teak (*Pericopsis elata*) and wild mahogany (*Tapirira guianensis*).

However, none of these or other works in the field of wood thermal modification specifically focus on the topic of color in connection with the chromophoric structures associated with it. At a constant heat treatment duration, an increase in treatment temperature determines the intensity of changes to a darker color, however, reported results provide ambiguous data as to whether and to what degree the extension of heat treatment duration at a constant temperature at the same time changes the color and properties of the wood [27]. An important carrier of color can be chromophores contained not only in the wood itself but in the case of tropical wood species to a greater extent in its extractive substances, too. Many works report correlations between color change and wood mechanical properties, but these dependencies are questionable in the case of wood species with a large content of different extractives, e.g., tropical wood species and black locust [2,21,22,28].

### Goals

The objective of this work is to investigate the color properties of six thermally modified tropical woods by different spectroscopic techniques in order to obtain a modeling of the changes validated by rigorous statistical methods together with principal component analysis. These results may contribute to the understanding of the chemical degradation mechanism of thermally modified wood by monitoring the changes in the extractive content yield and the development of relevant chromophores. Because the aesthetic aspect is often the prevailing criterion of a decision made by the end user, the color of thermally treated wood should be controlled during the production process, too. The results of this experiment should show certain trends in color development in the production process of thermally modified tropical wood species, which can be optimized with this in mind.

This new approach was applied for evaluating samples of Spanish cedar (*Cedrala odorata*), iroko (*Chlorophora excelsa*), merbau (*Intsia* spp.), meranti (*Shorea* spp.*),* padouk (*Pterocarpus soyauxii*), and teak (*Tectona grandis*), thermally modified at temperatures of 180 °C, 200 °C, and 220 °C, including the reference unmodified samples.

Other partial goals of this research leading to the main objective are

1. Determination of the influence of the thermal modification temperature on individual parameters (L*, a*, and b*) of the CIELAB color space, including total color difference (ΔE*) and on UV-Vis diffuse reflectance parameters, i.e., k/s intensity, yellowness (Y), and ISO brightness, of selected wood species.
2. Determination of the effect of the thermal modification temperature on individual parameters (L*, a*, and b*) of the CIELAB color space and yellowness index (Yi) of extractives isolated from these woods into acetone, ethanol, and ethanol-toluene and their mass yields (wt.%).
3. Quantitative and qualitative evaluation of extracted chromophoric compounds in the above-mentioned individual solvents using UV-Vis spectroscopy.
4. Statistical evaluation of the measured data of solid samples and extractives.

## 2. Materials and Methods

### 2.1. Materials

Six different tropical woods, which are summarized in Table 1, were used in this study [19,29,30].

### 2.2. Sample Preparation

#### 2.2.1. Thermal Modification

Radially cut samples with dimensions of 20 × 20 × 300 mm (r × d × l) were divided into 4 groups for each type of wood. One group was left as a reference (denoted as 20 °C) and the other three were intended for thermal modification at temperatures of 180, 200, and 220 °C. All samples were pre-conditioned in a Memmert climate chamber at a temperature of 20 ± 1 °C and a relative air humidity of 65 ± 3% to achieve an equilibrium moisture content of 12 ± 1%.

The thermal modification of the test specimens was carried out in a Katres thermal chamber according to the principle of the Thermowood^®^ method (Thermo S for 180 °C and Thermo D for 200 and 220 °C) in three phases [31]:

1. Rapid heating to a temperature of 100 °C followed by gradual heating to a temperature of 130–140 °C for about 3–4 h (heating step 8–10 °C per hour) depending on the type of wood, its thickness, and moisture content. During this phase, the wood was dried to a nearly 0% moisture content.
2. After reaching the required temperature (180, 200, and 220 °C), the thermal modification itself takes place for 3 h. Sensors control the constant temperature level of the samples’ cross-section. During heat treatment, water vapor was introduced to the chamber, which served as a protective medium against ignition and favorably influenced the ongoing chemical reactions.
3. The final stage is a cooling and regeneration process (moistening) lasting 5–15 h. By gradually watering the wood and dropping the temperature to 80–90 °C, the samples return to the desired moisture level of approximately 5–7%. The chamber was opened at a temperature of 40 °C to avoid thermal shock in the modified wood.

#### 2.2.2. Preparation of Wood Samples for Analyses

**Figure 1:**
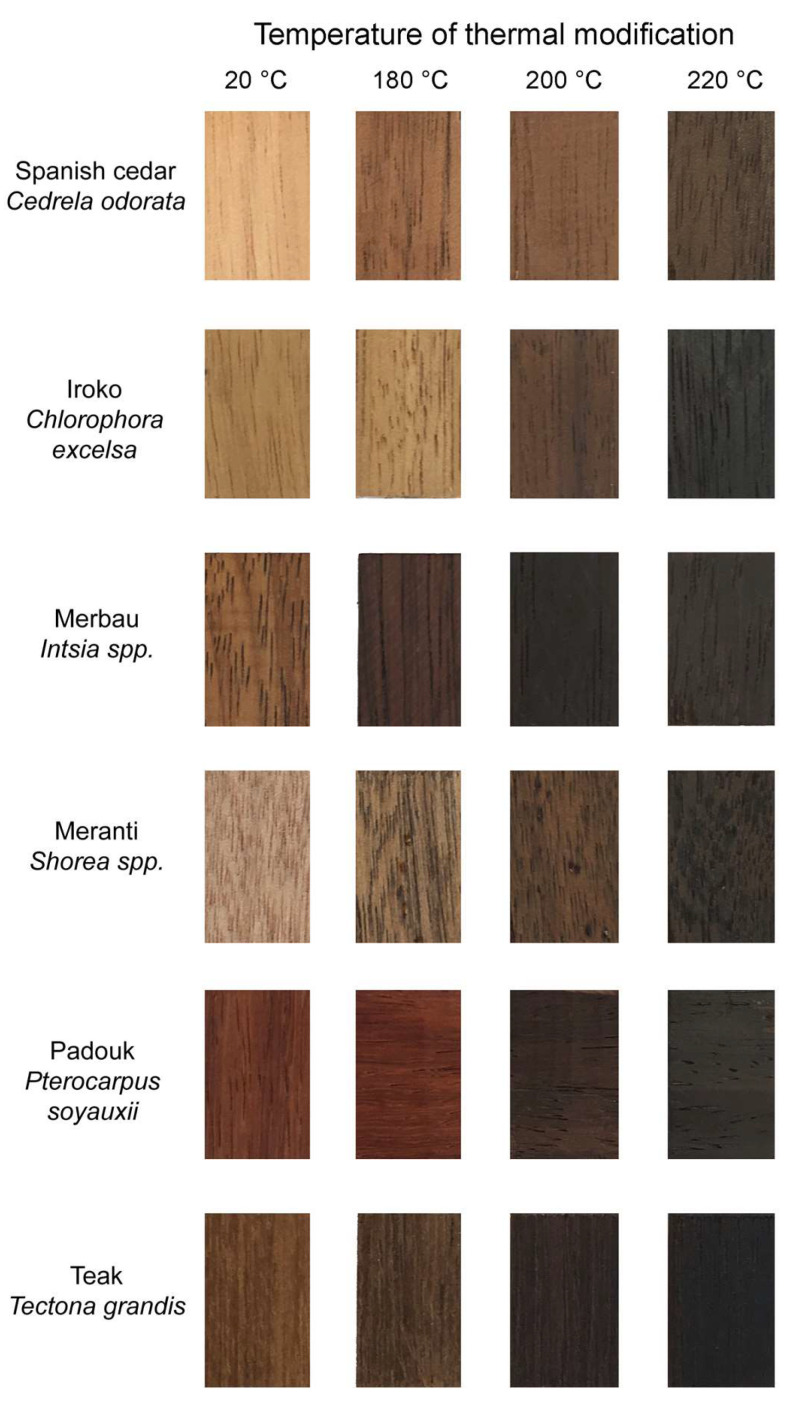
The appearance of reference and thermally modified samples at temperatures of 180, 200, and 220 °C.

From all reference and thermally modified woods, seven samples with dimensions of 3 × 20 × 30 mm were cut for color measurement of solid wood samples using a spectrophotometer device (Figure 1). After cutting, the samples were stored in darkness and conditioned at 65% relative humidity and a temperature of 21 °C to reach a 12% moisture content. The radial surface was used for the measurements.

Moreover, a part of each original tested series was also disintegrated using a knife mill (MF 10 BASIC, Germany) and then sieved through a standardized set of nets. To determine the extractives content, conditioned samples of the fraction 0.425–0.250 mm (40–60 mesh) were used. This wood sampling and preparation was carried out according to TAPPI T 257 cm^−2^ [32] and ASTM D 1107—96 [33] standard procedures. From each test series, 2 samples were prepared in this way, on which, the measurement took place.

### 2.3. Methods

**Figure 2:**
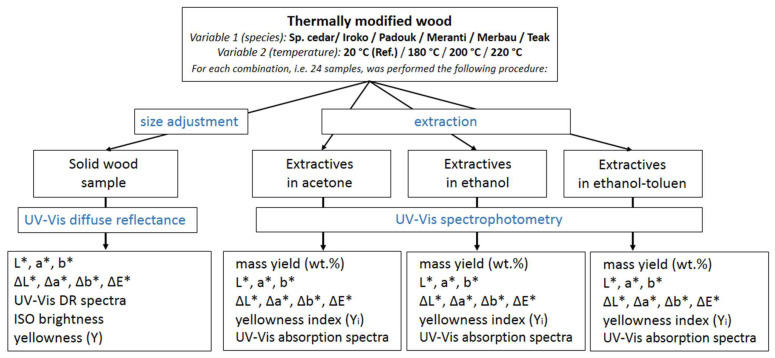
Design of an experiment for the analysis of color and chromophoric groups in thermally modified wood.

All experiments were performed according to the scheme in Figure 2.

#### 2.3.1. Measurement of Color Parameters Including UV-Vis Diffuse Reflectance Spectra of Solid Wood Samples

The characteristics describing the color of the sample (L*, a*, and b*) as well as the characteristics of diffuse reflectance SCI (specular component included), which are k/s parameters for 360–740 nm, yellowness Y, and ISO brightness were measured using a benchtop-type spectrophotometer Konica Minolta CM-5 (Konica Minolta, Osaka, Japan). The parameters ΔL*, Δa*, Δb*, and ΔE*, and brightness reversion were subsequently calculated from the measured data.

Before measurement, samples were conditioned at a room temperature of 23 ± 1 °C and a relative air humidity of 50 ± 2% according to standard ISO 187 [34].

The measurement of the color change took place according to the principle of spectral reflection with a wavelength resolution of 10 nm and a measurement diameter spot of 3 mm. The spectrophotometer was calibrated before each measurement according to the procedure recommended by the producer guided by a computer using the Color Data Software CM-S100W SpectraMagicTNNX (Konica Minolta, Osaka, Japan).

The color parameters according to ISO 11664-4 method [35] were determined for reference and thermally modified wood samples (for each series on seven replicates) by the CIELAB system, in which L* denotes lightness (from 0 black to 100 white), a* denotes color (−a green to +a red), and b* denotes color (-b blue to +b yellow). ΔL*, Δa*, and Δb* represent the differences in the individual coordinates of the color space before and after thermal modification. The total color differences ΔE* for individual samples were then evaluated according to ISO 11664-6 [36] and following Equation (1):

(1) $$\Delta E^{*}=\sqrt{{\left(\Delta L^{*}\right)}^{2}+{\left(\Delta a^{*}\right)}^{2}+{\left(\Delta b^{*}\right)}^{2}},$$

The reflectance spectra were measured in the wavelength range of 360 to 740 nm under illuminant D65 with specular components included. The 2° standard observer was set before the computation of color coordinates. The reflectance spectra were converted into k/s spectra using the Kubelka–Munk equation according to Equation (2) [37]:

(2) ks=1−R22R,

where *R* is the measured reflectance and *k* and *s* are the absorption and scattering coefficients, respectively. Four replicates were analyzed for each sample series and the resulting spectrum is an average of them. Yellowness and brightness values were provided directly by the instrument, Konica Minolta CM-5.

#### 2.3.2. Extraction

For the determination of extractive substances, the following solvents that are capable of extracting various substances from wood were chosen: acetone (C_3_H_6_O), ethanol (C_2_H_6_O), and ethanol-toluene binary mixture in a volume ratio 7:3 (C_2_H_6_O–C_7_H_8_).

The complete extraction method with acetone was performed according to TAPPI T 280 pm-99, Acetone Extractives of Wood and Pulp [38], and methods with ethanol (95%) and ethanol–toluene (7:3) were performed according to TAPPI T 204 cm-97, Solvent Extractives of Wood and Pulp [39]. The moisture content of each sample was determined prior to extraction according to TAPPI T 264 cm-97: Preparation of Wood for Chemical Analysis [40].

#### 2.3.3. UV-Vis Spectrophotometry Measurement of Extractives in Solvents

The analysis of samples of dissolved extractives in the liquid phase in individual solvents was carried out on a DR-6000 UV-VIS spectrophotometer (Hach, Colorado). The instrument was calibrated automatically on a cuvette with distilled water. First of all, the characteristics of used pure solvents were measured and then always subtracted from measured color parameters as well as spectra of the corresponding dissolved extract sample.

According to the CIELAB system, the characteristics describing the color (L*, a*, and b*) were determined for all samples (in two repetitions) and subsequently, the parameters ΔL*, Δa*, Δb*, and ΔE* were calculated. Measured data were evaluated similarly to the solid samples. Thus, the color deviation ΔE* was calculated using Equation (1).

Yellowness index (Yi) values were provided by the instrument DR-6000 at the wavelength of 720 nm. 

The absorbance spectra of samples in vitro were recorded in the wavelength range of 190 to 900 nm. At specific wavelengths, the following chromophoric structures were observed and evaluated: phenols at 460 nm, tannins at 700 nm, organics at 254 nm, volatile organic acids at 495 nm, and suspended solids at 810 nm.

#### 2.3.4. Statistical Evaluation

Statistical evaluation of the measured data of solid samples and extracts was performed using TIBCO Statistica software version 14 (TIBCO Software Inc., Palo Alto, CA, USA) for the indicated statistical methods: cluster analysis, principal component analysis (PCA), correlation (Pearson’s correlation coefficient), and regression analysis.

## 3. Results and Discussion

### 3.1. Measurement of Color Parameters of Solid Wood Samples Using UV-Vis Diffuse Reflectance

The color parameters of a* and b* acquire only positive values for all samples and their colors therefore have shades of red (a*) and yellow (b*) as shown in Table 2. Padouk wood, namely PA20 (a* = 29.00) and PA180 (a* = 26.53), is the most red-colored of all the samples. The a* value of the other reference tropical wood samples ranges from 9.81 to 14.77. Thermally modified samples IR220, PA220, TE220, and ME200 have the lowest values of parameter a* in the range of 2.91–4.24. Regarding the redness parameter, we can observe that its greatest decrease was recorded between 200 and 220 °C for all samples, except for merbau. There is no similar trend for brightness and yellowness. The samples of padauk (PA20, PA180) together with TE20 and IR180 have the highest values of the b* parameter (30.57–26.19). The average range of these values for the other reference samples is 17.91–24.93. The lowest values of the b* parameter (4.17–5.24) are again, as with the a* parameter, observed for samples IR220, PA220, TE220, and ME200. The SC20 sample (66.32) and also the MT20, IR20, and IR180 samples (L* > 50) have the highest brightness (L*). The brightness of the other reference samples is within a narrow range of 39–41. The lowest brightness values (L* < 20) are shown by samples IR220, ME220, TE220, MB200, and TE200. L* values clearly decrease with the increase in thermal treatment for all samples of tropical woods compared to the reference sample. This trend was also observed in the work of many other authors [41,42,43,44] investigating other wood species. Monaco et al. [41], who have studied the effect of thermal treatment on chestnut wood (*Castanea sativa* Mill.) at temperatures of 140, 170, and 200 °C, moreover observed that ΔE values, calculated for the three thermal treatments in respect to untreated samples, depend mainly on L* variations in the case of their samples because the chromatic coordinates a* and b* did not change significantly except for an increase clearly observed at 170 °C and corresponding to an increase in both yellow and red components.

**Figure 3:**
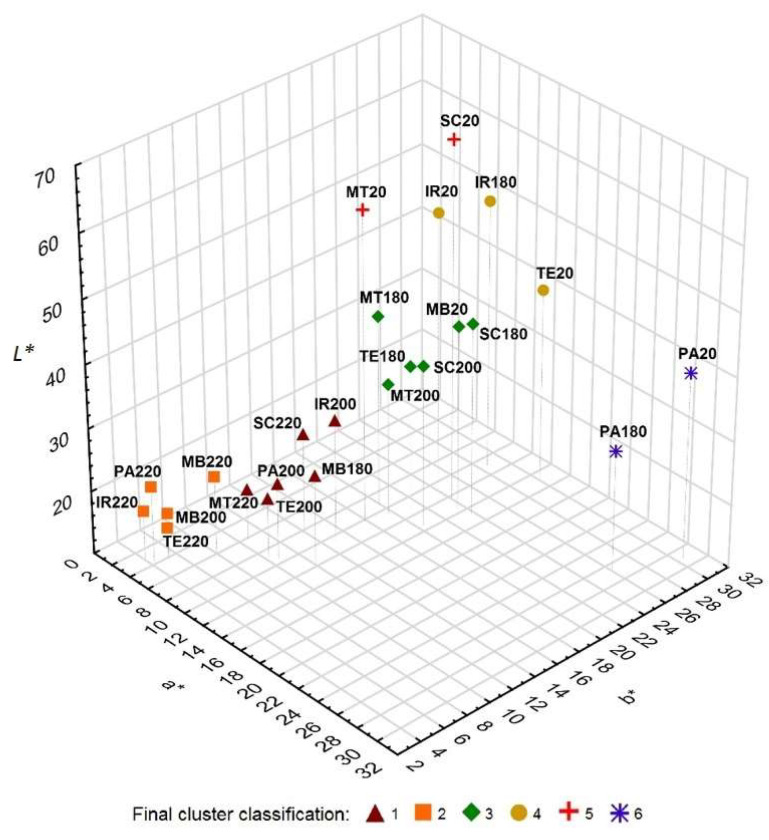
A 3D graph of individual L*, a*, and b* values of the color space defining the color of selected types of thermally modified wood samples—cluster analysis.

In the next step, a cluster analysis (Figure 3) was performed for the parameters L*, a*, and b*, which divided all samples into six clusters according to similarity. The first group includes six thermally modified samples, MT220, SC220, PA200, TE200, IR200, and MB180. The second cluster consists of samples modified at higher temperatures, PA220, TE220, IR220, MB220, and MB200. The third cluster includes MT200, MT180, SC200, SC180, TE180, and MB20. It is interesting that this group also includes the merbau reference sample, which has similar optical properties to thermally modified samples of other woods at temperatures of 180 °C and 200 °C. At the same time, there is twice the similarity of two samples of the same wood and at different modification temperatures (MT200 and MT180, and SC200 and SC180). The fourth cluster includes IR180, IR20, and TE20, and the fifth includes MT20 and SC20. The samples of both clusters differ from the others by a higher L* value and from each other mainly by the values of a* and b*. The last cluster consists of samples PA20 and PA180 with a high redness value.

**Figure 4:**
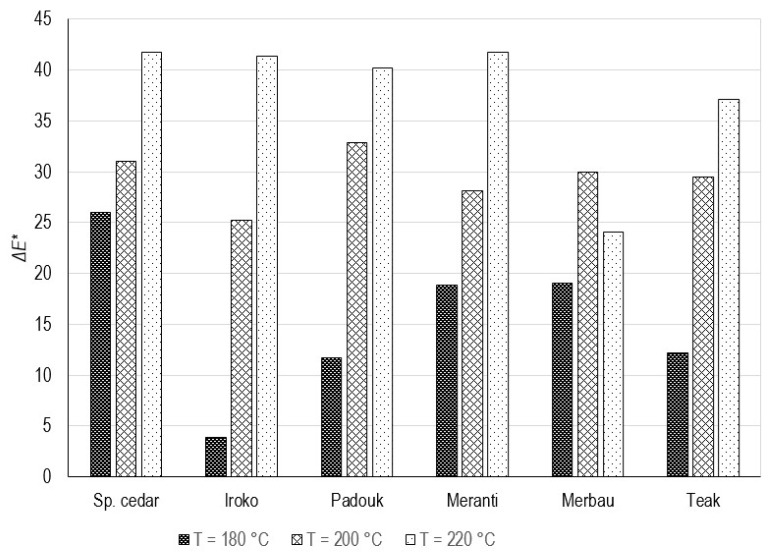
Total color difference (ΔE*) of selected types of thermally modified tropical wood samples at temperatures of 180, 200, and 220 °C.

The graph in Figure 4 shows the development of the average values of the calculated total color difference (ΔE*), i.e., the total color change compared to the respective reference samples. In all cases, except for ME220, there is an increase in ΔE* along with an increasing thermal modification temperature. In the case of merbau, the highest ΔE* is at 200 °C, and a decrease is noted at 220 °C. A temperature of 180 °C had the greatest impact on the overall color change for SP180 (ΔE* = 26) and the least for IR180, which remained without a significant color change (ΔE* = 4). At a temperature of 200 °C, the largest color change occurred at PA200 (ΔE* = 33) and the smallest again at IR200 (ΔE* = 25.5). It can be said that the ΔE* caused by the temperature of 200 °C of most samples, except iroko, is very similar (32.90–28.15), and compared to the other temperatures, the smallest fluctuations in values are observed within this group. The highest modification temperature of 220 °C caused color changes at a similar level (ΔE* ≈ 40–42) for SC220, IR220, PA220, and MT220. ME220 was relatively least affected by this temperature (ΔE* = 24). The most significant increase in ΔE* occurred in the case of SC, MT, and MB during thermal modification from 20 °C to 180 °C. For IR, PA, and TE, this increase was the highest from 180 °C to 200 °C.

The measured yellowness (Y) of the samples is a number calculated from the spectrophotometric data and indicates the degree of deviation of the wood color from colorless or the preferred white towards yellow (ASTM E313 [45]). ISO brightness indicates the actual percentage of light reflected from the wood sample at 457 nm (Tappi North America, 2017). In general, within one series of samples, both parameters decrease with increasing modification temperature. Small deviations from this trend, i.e., a slight increase in these values compared to a lower modification temperature, are observed in the case of ISO brightness for samples IR180, TE180, PA200, PA220, and MB220, and in the case of yellowness for samples IR180 and MB220. Determined values of ISO brightness and yellowness listed in Table 2 were further included in the statistical evaluation using a principal component analysis (PCA).

The thermal modification temperature and wood species are the most important factors that affect the final color. When the temperature is higher, the wood color is darker [46]. Like for Baar and Gryc [47], it was also confirmed that lighter wood is more susceptible to changes in surface color and brightness, and dark wood shows the smallest deviations. In general, there is an increase in the parameter ΔE* as the temperature of thermal modification increases, as in the studies published by Gaff et al. [22] for padouk merbau, mahogany, and iroko wood, Cuccui et al. [48] for teak wood, or Kačík et al. [28] for acacia. The strong correlation between the lightness difference value (ΔL*) and the content of hemicelluloses was moreover published by Gaff et al. [22]. Such changes also increase ΔE* [49,50] relative to untreated wood.

### 3.2. Measurement of UV-Vis DR Spectra and Quantitative Evaluation of Chromophoric Groups in Solid Wood Samples

**Figure 5:**
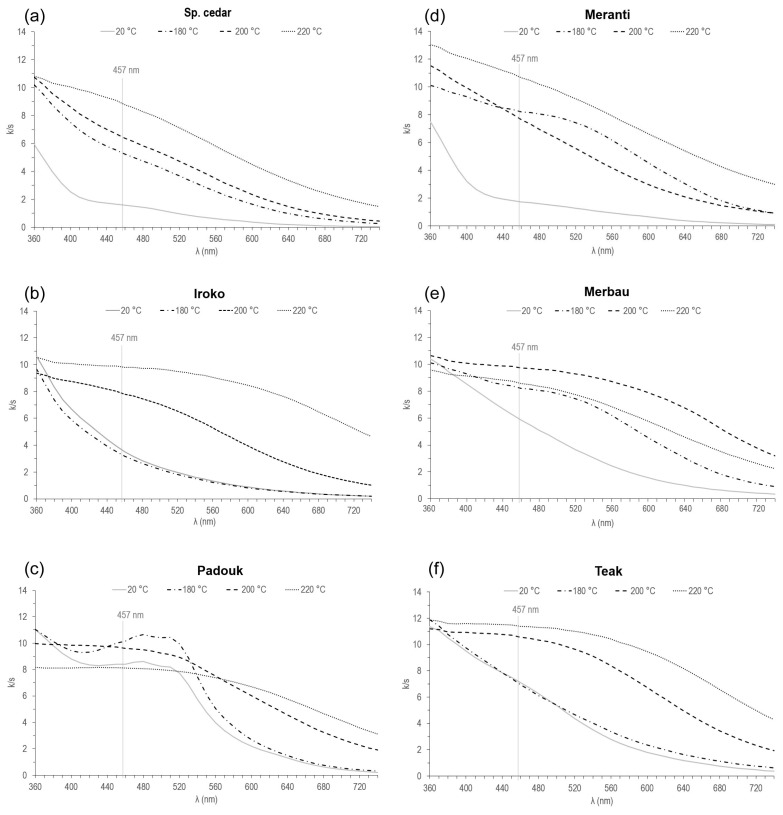
UV-Vis DR spectra of selected tropical wood samples. (**a**) Sp. cedar, (**b**) iroko, (**c**) padouk, (**d**) meranti, (**e**) merbau, and (**f**) teak thermally modified at temperatures of 180, 200, and 220 °C compared to reference unmodified samples.

UV-Vis diffuse reflectance spectra of individual tropical wood samples thermally modified at temperatures of 180, 200, and 220 °C, including a comparison with the reference sample (20 °C), are presented in Figure 5. k/s values, which in the range of this measurement (360–740 nm) indicate the most frequent occurrence of chromophoric groups [5], were monitored and used for their quantitative evaluation. The course of recorded spectra was also evaluated.

The absorption coefficient in the UV region at 360 nm corresponds to the chromophoric structures containing phenolic aldehydes (coniferaldehyde), unsaturated ketones, quinones, stilbenes, other conjugated carbonyl structures, and charge transfer complexes [51,52]. It is known that lignin has only a small contribution to absorption in the visible wavelength region but increases rapidly with decreasing wavelength into the ultraviolet regions [5].

The absorbance in the visible region of blue and violet color (380–520 nm) is recognized as a yellow color. In the visible region of measured spectra, there were no significant absorption bands (except PA180), which would indicate the presence of chromogens at the chromaticity centers. The absorption coefficient at 440 nm can be attributed to the formation of colored quinoid compounds originating not only from the degradation and oxidation of the aromatic hydroxyl groups of lignin but also from aromatic extractives upon heat treatment [53]. The absorption coefficient of all samples (except IR180, TE180, and PA220) after thermal modification at this wavelength is always higher than that for the corresponding reference sample. Equally, the brightness of all thermally modified wood (except the same samples mentioned above) is always lower in comparison with that of the reference wood (note the k/s values at 457 nm).

Similar to the case of Sp. cedar (Figure 5a), it can also be observed for meranti that the value of k/s increases with the increasing temperature of thermal modification and decreases with increasing wavelengths. In addition, the spectra of SC20 and MT20 have very similar courses and k/s values. At 360 nm, the k/s intensity of all thermally modified SC samples is 1.7–1.8× higher than that of SC20, which indicates an increase in the content of chromophoric groups.

For iroko (Figure 5b), the intensity of the k/s spectra does not always increase with temperature, such as for SC and MT. For IR180, there was a decrease in this intensity, even below the values of IR20 in the entire measurement area. The most significant increase in k/s intensity in this group was observed at 457 nm for sample IR200 (2.4× compared to IR180) and sample IR220 (another 1.2× compared to IR200). In the case of iroko, it can be observed that the k/s values of all four samples in the 360 nm region are very close (9.4–10.6). Similar clustering is also evident in the merbau (9.6–10.6) and teak (11.2–11.9) series.

In the case of padauk (Figure 5c), in the range of wavelengths from 360 to 580 nm, it is not possible to talk about a specific relationship between the intensity of the k/s spectra and the modification temperature, since there is an intermingling of the individual spectra. The course of the spectrum of PA220 (descending and slightly convex) is particularly interesting. Its intensity is the lowest at 360 nm (8.2), compared to the other measured spectra in this group, and remains at this intensity approximately until the wavelength of 520 nm, when the PA20 spectrum is below its level, and from 535 nm, when the PA180 spectrum is also below its level. It is also worth noting the non-standard deviation of the PA180 spectrum in the range of wavelengths 440–540 nm, when its intensity exceeds the spectra of samples modified at higher temperatures. A similar deviation in the same range of wavelengths, but to a lesser extent, is observed in the spectrum of PA20.

MT220 (Figure 5d) achieves the highest k/s values of all sample types at wavelengths of 360 nm (13.1) and 392 nm (12.2).

In the case of MB200 (Figure 5e), k/s values of the monitored wavelengths (360, 440, and 457 nm) are at a very similar level (ca. 10). The same behavior and a low spread of values around 10 is observed in the case of the IR220 and PA200 spectra, too. The spectrum of MB220 is abnormally located below the other spectra of samples from this given series up to a wavelength of approx. 385 nm. Again, a certain similarity with sample PA220 is obvious.

TE220 (Figure 5f) is unique with the highest value of k/s (11.4) at a wavelength of 457 nm. The highest increase in the intensity of k/s spectra at a wavelength of 457 nm is between samples TE180 and TE200 (by 3.5). For all samples in the teak series, very similar trends appeared for iroko samples, too.

The apparent absorption changes in spectra of investigated samples after thermal treatment suggested the generation of some types of chromophores in the degradation, condensation, and oxidation progress, which explains the samples yellowing and their loss of brightness after the action of elevated temperature. However, it is very difficult to differentiate structural differences between these chromophores, because the nature of color change is complex and their composition depends on the extraction method used [54]. Considering that tropical woods have a high content of extractive substances, it can be assumed that thermal oxidation processes play a significant role in this case. Bekhta and Niemz [55] have reported that the color changes caused due to elevated temperatures can be because of the formation of secondary condensation products and/or degradation products, quinoid substances, for instance, which are known to be intensely colored. Keating et al. [52], as well as Chen and Li [53], add that the reactive compounds may include degradation products from the cleavage of α- and β-aryl ether bonds in lignin and degradation products from hydrolyzed hemicelluloses [56,57], too.

### 3.3. Determination of the Extractive Content in Wood

Wood extractives are natural products extraneous to a lignocellulose cell wall. They are present within a cell wall but are not chemically attached to it. It is known that some tropical wood species have high extractive contents in the heartwood [58]. These extractives influence the color, odor, durability, and technological properties of the different wood species [59]. In general, the amount of lipophilic fatty acids in tropical wood species compared to softwood and hardwood is very low [60]. From a group of hydrophilic constituents, many phenolic compounds (i.e., phenolic acids, flavonoids, sterols, stilbenes, tannins, and lignans), terpenes and terpenoids, free sugars, and sugar alcohols can be detected [61,62]. Some of the most volatile compounds produced during the thermal modification of wood are released, e.g., fats, waxes, furfural, and hydroxymethylfurfural [63], and on the contrary, new extractive substances are created by the degradation of the main components of wood, e.g., dehydration and degradation products of polysaccharides and phenolic compounds like catechol, vanillin, vanillic acid, 3-vanillyl propanol, and coniferyl aldehyde, probably resulting from lignin or phenolic extractives [64].

These remaining substances in the wood can be extracted by several solvents. Polar solvents penetrate well into the cell wall, due to which the cell wall partially swells. Organic salts, oligosaccharides, and carbohydrates can be specifically extracted from wood by water (hot or cold). Ethanol removes the tannins, dyes, or glucosides. Acetone extracts mainly the fatty and resinous acids and sterols. Non-polar solvents (e.g., benzene, petroleum ether, ether, and toluene) extract the fats, fatty acids, and their esters, resins and resin acids, waxes, and sterols from wood. The advantage of using an ethanol–toluene mixture is its non-carcinogenicity compared to benzene. Using this mixture, it is possible to extract well from the wood most phenolic substances (lignin fraction, sterol, tannin, and plobaphen), some organic acids (resin acid, amino acid, vanillic acid, and syringic acid), and other substances such as pigments and syringaldehyde [65,66].

Table 3 lists the percentage mass representation w (wt.%) of individual extractive substances soluble in acetone, ethanol, and ethanol–toluene binary mixture in thermally modified and reference tropical wood samples, including parameters describing their optical properties (ΔE*—total color difference; Yi—yellowness index) from the next chapter.

**Figure 6:**
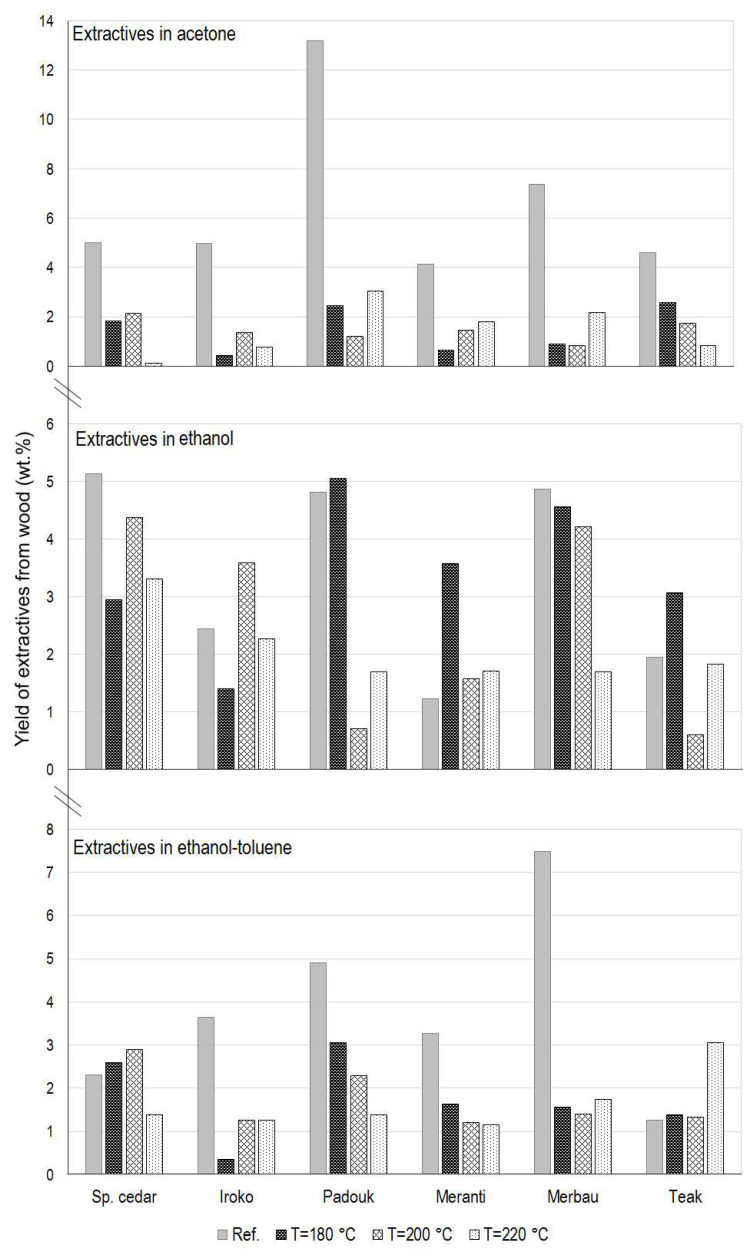
The average amount of extracted substances in acetone, ethanol, and ethanol–toluene from thermally modified and reference samples of selected tropical woods.

In the following graphs in Figure 6, we can see even better the average percentage by weight of the extracted substances from individual samples in the three selected solvents.

Upon closer analysis, it is evident that in the case of the six reference samples, four of them (IR20, PA20, MT20, and TE20) had the greatest gain in extractive substances when acetone was used. The greatest gain for samples thermally modified at 180 °C was, in all six cases, when ethanol was used. For samples modified at higher temperatures (200 and 220 °C), in most cases, ethanol was the most effective during extraction (except for padauk and teak, when acetone or a binary mixture of ethanol–toluene was more efficient).

Specifically, the greatest amount of substances extracted into acetone was obtained for PA20 (13.2 wt.%), even in comparison with other samples extracted in other solvents. The lowest amount of extractives across all samples and solvents (0.1 wt.%) can be observed in SC220 in the case of acetone, which is approximately a twenty-fold decrease compared to the SC200 sample. The amount of extractive substances in samples SC220, IR220, and TE220 is below the limit of 1 wt.%, and compared to the modification at a temperature of 200 °C, their representation decreases. On the contrary, for PA220, MT220, and MB220, an increase in the obtained extractives was observed when the temperature increased from 200 to 220 °C. 

The greatest amount of substances soluble in ethanol was obtained for samples SC20 and PA180 (both 5.1 wt.%), MB20 (4.9 wt.%), and PA20 (4.8 wt.%), and the lowest for TE200 (0.6 wt.%) and PA200 (0.7 wt.%).

In the case of extraction into ethanol–toluene, MB20 shows the highest value of the representation of extractive substances and, in comparison with other types of wood and concerning the solvents used, it has the second highest yield. On the contrary, the lowest amount of extractives soluble in this solvent (0.35 wt.%) was obtained from the IR180 sample, when compared to IR20, there was a roughly ten-fold decrease. In addition, as can be seen from the graph in Figure 6c, an interesting group of 13 samples is observed during extraction into this binary mixture, from which a very similar amount of extractives of around 1.3 ± 0.5 wt.% was obtained.

The color stability of heat-treated wood during artificial weathering has long been the subject of much research, as wood treated in this way tends to lose color, especially on the exterior by the action of light, or just by the leaching of extractive substances and degradation products, which can be carriers of chromophoric structures [67,68,69]. Although many improvements have been made, this issue is still a challenge. In future research, it is therefore necessary to look for new technologies for color stability of heat-treated wood using new surface treatments (varnishes, metal oxides, nanoparticles, etc.) [41].

### 3.4. In Vitro Color Measurement of Extractive Compounds Using UV-Vis Spectrophotometry

The color characteristics (ΔE*, respectively, L*, a*, b*, and Yi) of the individual extractive samples are not further discussed separately, but they were used for statistical evaluation in the PCA analysis.

The absorbance spectra of all investigated samples in vitro were recorded in the wavelength range of 200 to 900 nm. The spectrum of the respective solvent was always subtracted from the spectra of the dissolved extractive samples. At specific wavelengths, depending on the extractive solvent, the following chromophoric structures could be observed: organic acids at 254 nm, phenols at 460 nm, volatile organic acids at 495 nm, tannins at 700 nm, and suspended solids at 810 nm. The course of the recorded spectrum was evaluated, too.

**Figure 7:**
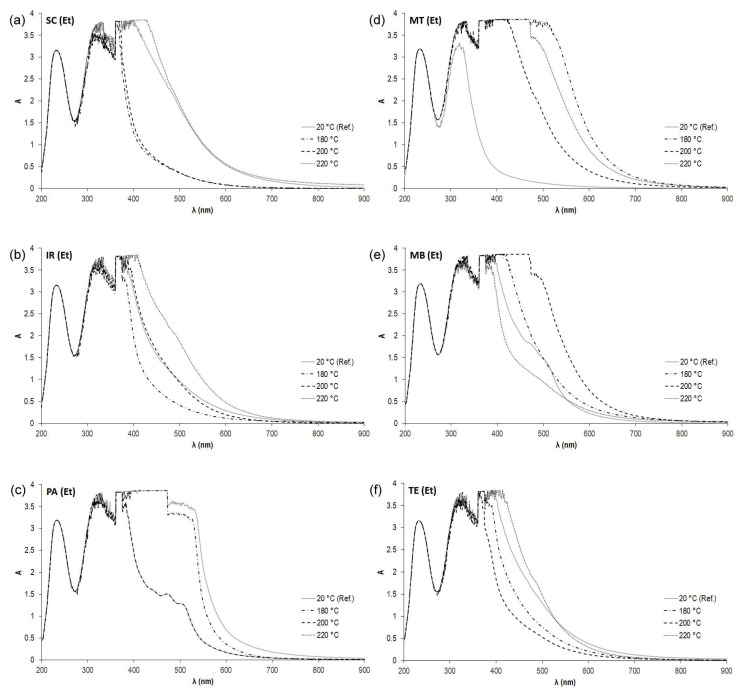
Absorption spectra of ethanol extractives from selected tropical wood samples. (**a**) Sp. cedar, (**b**) iroko, (**c**) padouk, (**d**) meranti, (**e**) merbau, and (**f**) teak thermally modified at temperatures of 180, 200, and 220 °C compared to reference unmodified samples.

The most effective solvent in the case of thermally modified samples (mainly at a temperature of 180 °C), which extracted the highest percentage of compounds from wood, was ethanol. The high content of ethanol extractives is because it is a more polar solvent than acetone that can dissolve the most represented polyphenols [70]. For that reason, the absorption spectra of the extractives dissolved in ethanol were also analyzed and are presented in Figure 7.

In dissolved extractives, light is mainly absorbed by lignin below 500 nm and by phenolic extractives, such as tannins, flavonoids, stilbenes, and quinones, above 500 nm [71]. The discoloration reactions that occur during heat treatment are often justified by the formation of colored oxidation and degradation products involving the cell wall constituents and extractives located in cell vacuoles [50,72].

#### 3.4.1. The Evaluation of the Measured Spectra in the Region below 500 nm

Thermally modified samples at the highest temperature of 220 °C showed the highest absorption in the respective series in the cases of SC220, IR220, and TE220. On the contrary, the lowest absorption associated with a decrease in volatile organic acids and phenols compared to the standard samples was observed in the cases of the PA220 and MB220 samples. For SC, IR, PA, and TE samples thermally modified at temperatures of 180 and 200 °C, the measured absorbance in these areas was lower than in the case of the respective reference samples. The spectrum of the reference sample PA20 is the only one that shows the highest intensity in the region below 500 nm compared to the other thermally modified samples from its series. On the contrary, the reference sample MT20 shows the lowest intensity in the entire measurement area.

For all samples, the highest absorption bands are observed in the wavelength range around 460 nm, where an increase in the phenolic hydroxyl group can be detected. As an auxochrome group, it could also contribute to the darkening of the thermally treated wood.

The increased absorption band at approx. 495 nm suggests the release of acidic substances, specifically volatile organic acids (acetic and formic acid), after the degradation of acetyl groups in polyose [73]. Acids can catalyze the condensation and degradation reactions in lignin structures [49] and extractives after heat treatment in the presence of water and contribute to the discoloration of the wood.

#### 3.4.2. The Evaluation of the Measured Spectra in the Region above 500 nm

It has been established that around wavelengths of 700 nm, a mixture of extractable components is detected, such as tannins and their derivates, e.g., ellagitannins due to their polymerization by oxygen and increased temperatures [74,75]. Absorbance in the region of these wavelengths, and therefore an increased amount of corresponding compounds, is observed in samples of padouk (PA20, PA180), meranti (MT180, MT200, and MT220), and merbau (MB200). An increased amount of other suspended solids compared to the other samples is detected in the 810 nm region for the SC220 and PA20 samples.

### 3.5. Extractive Substances Determined in Tropical Wood Species by Other Authors

Tenorio et al. [76] published, in their work focused on Spanish cedar, a high value of soluble extractives in an ethanol–toluene mixture found in the 4- and 5-year-old trees, indicating a high presence of waxes, fats, resins, phytosterols, nonvolatile hydrocarbons, and polar extractives in the wood of this species. Among the water-extractable substances were tannins, gums, sugars, and coloring matters and starches. Likewise, high levels of soluble extractives in NaOH indicate a high presence of low-molecular-weight extractives, such as carbohydrates, consisting mainly of hemicellulose and degraded cellulose in wood.

Biwôlé et al. [77] identified chlorophorin, a highly hydrophobic substance, in iroko powder before and after 30 days of immersion in cold water. They also determined that cudraxanthone I and geranyl trihydroxystilbene exhibited low hydrophobicity in wet conditions. Biwôlé et al. [78] published that iroko wood is rich in polyphenols and used MALDI-TOF analysis instead of the abovementioned other compounds such as geranyl-trihydroxy-stilbene and neocyclomorusin.

The main extractive of padouk is homopterocarpin, directly recovered with a good purity via acetone extraction. It also contains pterocarpin and anethole, C5 and C6 carbohydrates, C14–C18 fatty acids, principally hexadecanoic acid, and triglycerides [53].

Yasuda et al. [79] studied the effect of meranti extractives on the manufacture of plywood. Using ^1^H and ^13^C NMR spectroscopy, they detected a high content of phenolic compounds, namely secondary oxidative polymers of resveratrol (e.g., hopeaphenol) and its related stilbenes. Other extractives of *Shorea* species, chrysophanol, scopoletin, and hexamethylcoruleoellagic acid, are also known [80].

Specifically, Kilic and Niemz [81] detected fatty acids, 3-hydroxybenzoic acid, vanillic acid, 3,4,5-trihydroxybenzoic acid, 4,4′-dihydroxy-3,3′-dimethoxystilbene, sitosterol, and sugars in merbau using GC/MS analysis. Malik et al. [82] state that merbau extractives are a promising material for the enhancement of wood properties because of their high content of phenolic compounds, particularly resorcinol.

Myo Aung [83] characterized the teak methanol extractives via HPLC and detected 2-methyl anthraquinone (tectoquinone), the compound responsible for the durability, i.e., mainly insecticidal properties, of teak wood along with a variety of extractives in lower quantities. The hydrophobicity, antioxidant properties, and oily nature of teak wood were mainly due to the caoutchouc compounds [84,85].

### 3.6. Statistical Evaluation

#### 3.6.1. Principal Component Analysis (PCA)

**Figure 8:**
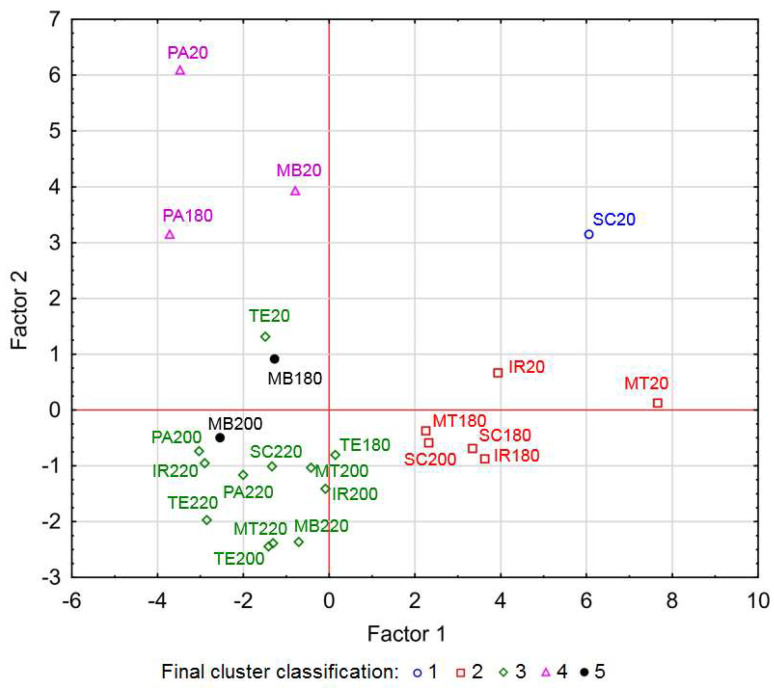
Projection of individual wood samples into the coordinate system of the 1st and 2nd principal components.

The values of all variables for wood and extractives (except ISO whiteness and all calculated parameters, ΔL*, Δa*, Δb*, and ΔE*) were taken into consideration from Table 2 and Table 3, from which the principal components were created. The original data were transformed into principal component space using PCA. Based on the results of the statistical processing, the number of components was chosen to be three, with the first component explaining 43.26%, the second component 20.94%, and the third component 8.15% of the variability. The dependence of the two most important components (1 and 2) can be seen in Figure 8.

When evaluating the contributions of individual variables to the principal components, it can be observed that the L* values and yellowness indices for wood (Y) and extractives (Yi), as well as the parameters a*(Ac) and b*(Ac) of extractives soluble in acetone have the greatest influence on component 1. The amount of all extractive substances w(Ac), w(Et) and w(Et-To) obtained from wood and the parameters a* and b* of the wood samples have the greatest influence on component 2. And the principal component 3 is influenced mainly by extractive substances soluble in ethanol, i.e., by the amount w(Et) of these extractive substances and the parameters L*(Et), b*(Et), and Yi(Et), and again by the parameters a* and b* of the wood samples.

**Figure 9:**
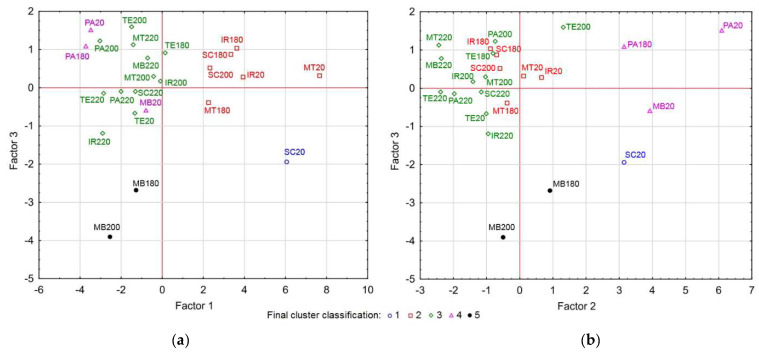
Cluster analysis shown in 1st and 3rd (**a**) and 2nd and 3rd (**b**) principal component spaces.

A cluster analysis was subsequently performed on these three selected components. The method identified five clusters. The distribution of the clusters is shown in the graphs in Figure 9. Each graph shows a different combination of two out of the three principal components against each other, as some clusters are distinguished from each other only in their specific combination (e.g., cluster 5 is not distinguishable from cluster 3 in the space of the first and second principal components in Figure 8, but what makes it different are the coordinates in the direction of the third component, as can be seen in Figure 9).

The first cluster consists of only one sample, which represents SC20. The second cluster includes six samples namely SC180, SC200, IR20, IR180, MT20, and MT180. The third cluster has the most samples and includes SC220, IR200, IR220, PA200, PA220, MT200, MT220, MB220, TE20, TE180, TE200, and TE220. The fourth cluster consists of three samples, namely PA20, PA180, and MB20. In the fifth cluster, there are the samples MB180 and MB200.

Based on this distribution, it can be seen that clusters 1, 2 (except SC200), 4, and 5 (except MB200) mainly consist of reference samples, where variability is obvious, and samples thermally modified at the lowest temperature, i.e., 180 °C. Conversely, cluster 5 includes all samples of thermally modified wood species at the highest temperature of 220 °C, most of the samples modified at 200 °C (except for the aforementioned SC200 in the second group and MB200 in the fifth group), and in addition, two teak samples (TE20 and TE180). This confirms the great similarity or homogeneity of color and related parameters of thermally modified tropical wood species starting at 200 °C, which were included in the cluster analysis.

If we approach the result of the cluster analysis from the point of view of individual wood species, the Spanish cedar samples are distributed in three clusters. SP20 is different to the others and no similarity is observed with any of the 24 samples (reference and thermally treated). Samples SC180 and SC200 show some similarity to iroko and meranti (reference and thermally treated samples at 180 °C). The SC200 sample belongs to the most numerous group 3, which also includes other thermally modified types of wood at the highest temperatures. A great similarity in optical properties can be seen in the iroko and meranti samples. As mentioned above, their reference and thermally modified samples at 180 °C are included in group 2, and the thermally modified samples at the two higher temperatures belong again identically in group 3. For PA20 and PA180, a certain agreement with MB20 (group 4) can be found, and the samples thermally modified at the two higher temperatures belong again to group 3. The merbau samples behave differently in terms of optical properties and, like the Sp. cedar, are distributed in three clusters, namely as follows: the reference sample MB20 together with PA20 and PA180 in a separate cluster 4, samples MB180 and MB200 in a separate cluster 5, and MB220 in the most numerous group 3. Teak is specific in that all samples from its series (TE20, TE180, TE200, and TE220) are assigned to cluster 3, which is typical for wood samples modified at the highest temperatures.

#### 3.6.2. Functional Dependence of Yellowness

**Figure 10:**
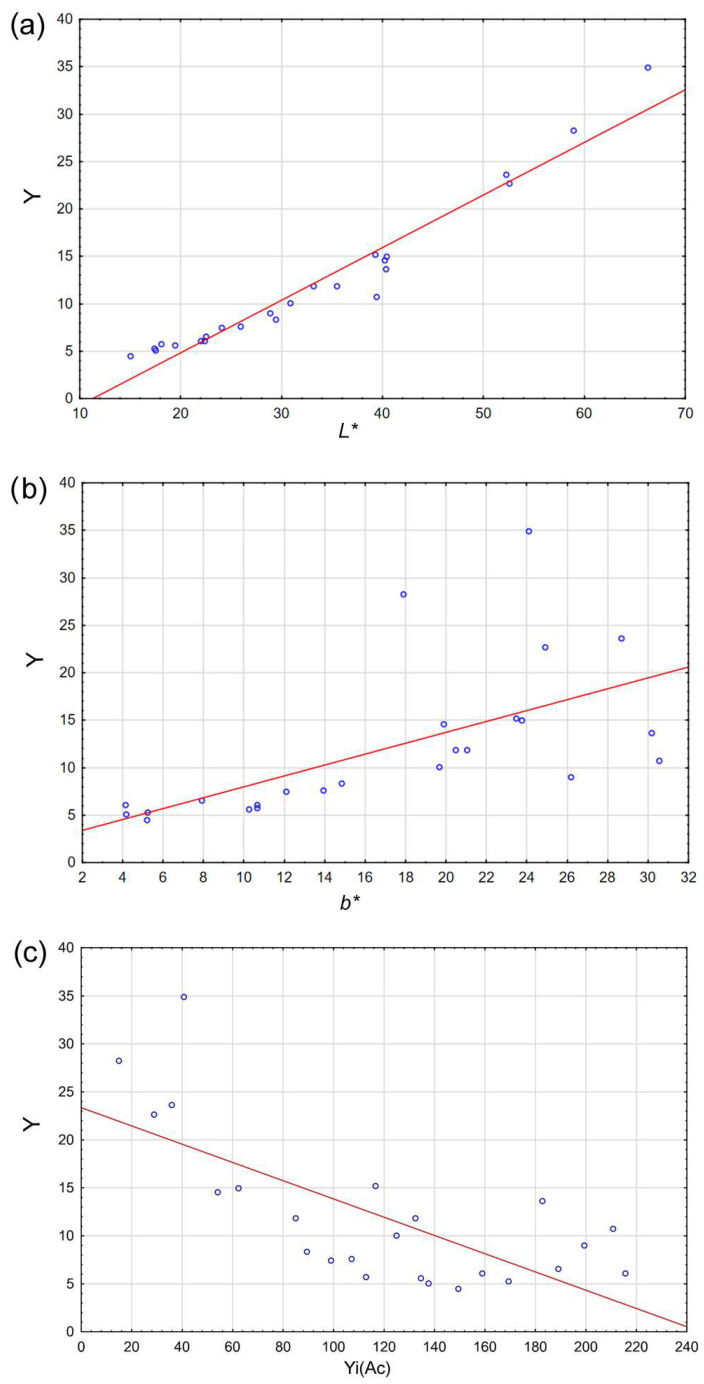
Relationship between wood yellowness (Y) and (**a**) L* parameter, (**b**) b* parameter, and (**c**) yellowness index of extractive substances soluble in acetone Yi(Ac).

The measured yellowness of solid wood samples (Y) is an important evaluation parameter for thermally modified wood. It can be seen from the correlation analysis that the most correlated quantity is L* with the value of the correlation coefficient r = 0.972 (Table 4). This value is statistically significantly non-zero and indicates a very high dependence. A specific comparison of the dependence of Y and L* is shown in Figure 10a.

When the functional dependence of wood yellowness (Y) on other variables is investigated using a regression analysis, then the only significant parameters are L* and b*. The regression model of this dependence is presented in Table 5. The coefficient of determination R2 is 0.973, which indicates a very high degree of explanation of the variable Y, with the help of parameters L* and b*. In other words, if we know the values of L* and b*, the value of Y can be estimated quite accurately using the regression equation. The dependence of Y and b* is shown in Figure 10b.

In search of connections between the yellowness of wood (Y) and the yellowness indices of the individual extractives Yi(Ac), Yi(Et), and Yi(Et-To), a regression analysis was performed, where Y was the dependent variable and individual yellowness indices were predictors. The results of this analysis are presented in Table 5. From the results, it can be seen that the only statistically significant factor is the yellowness index Yi(Ac). Even if we leave the insignificant factors Yi(Et) and Yi(Et-To) in the model, the coefficient of determination is only 0.510 compared to the previous analysis including the connection with L* and b*, i.e., 0.973. For completion, the relationship between Y and Yi(Ac) is also shown on the graph in Figure 10c, which is described by the equation Y = −0.0951 × Yi(Ac) + 23.3485.

## 4. Summary and Conclusions

The results have contributed to the understanding of the chemical degradation path of thermally modified tropical wood species by monitoring extractive content transformations in connection with their changes in color and the color of the wood itself. The importance of optimizing the treatment (temperature and duration of thermal modification or subsequent treatment related to increasing the stability of wood color in exterior conditions) for each wood species to make the best utilization of this material has been pointed out.

Solid Wood Samples

- The values of the L*, a*, and b* parameters are in all cases positive, and with increasing temperature of thermal modification, they have a decreasing trend—the samples lose brightness and darken. The exception is merbau wood, where these values increased between 200 and 220 °C.
- Based on the cluster analysis in the CIELAB color space, the samples can be divided into six clusters according to similarity. A special group is made up of padauk samples PA20 and PA180, distinguished by their significant redness (a* ≈ 26.5–29.0).
- As the thermal modification temperature increases, ΔE* increases for most samples except for merbau with the highest ΔE* value for sample MB200 (for MB220 this value decreases by almost 20%). At a thermal modification temperature of 200 °C, all samples (except IR200) have similar ΔE* values and at 220 °C, Spanish cedar, iroko, padouk, and meranti.

Extractive Samples

- In the case of the reference samples (except for SC and MB), the largest amount of extractives was obtained during extraction into acetone, into which mainly fatty and resinous acids and sterols are released.
- In the case of samples modified at higher temperatures, the highest yield of extractives (mainly phenolic compounds, glycosides, and dyes) was usually obtained using ethanol. Their representation in wood was mostly highest at a thermal modification temperature of 180 °C and decreased at 200 and 220 °C.

Assessment of Chromophoric Structures

- The increasing amount of chromophores with the thermal modification temperature is observed for Spanish cedar and meranti.
- The k/s intensity decreases along with increasing wavelength for all woods (except padouk).
- Padouk, merbau, and teak, and partially also iroko, modified at temperatures of 200 and 220 °C contain a comparable amount of chromophoric compounds detected at wavelengths of 360 nm and 457 nm.
- The discoloration of wood during thermal modification is due mainly to condensation and oxidation reactions. Both of these lead to the formation of extensive conjugation structures (alkenes and aromatics) while increasing the number of carbonyl or carboxyl groups.
- New types of extractive substances formed from hemicelluloses (2-furaldehyde, etc.), cellulose (lactones, etc.), and lignin (formic acid, methanol, monomer derivatives of phenols, etc.) are created already below a temperature of 180 °C when they are not subject to even more condensation reactions.

Statistical Evaluation

- Five clusters based on the similarity of the included parameters (L*, a*, and b* of solid samples and extractives, yellowness of wood, yellowness indices of individual extractives, and intensity k/s at selected wavelengths and amount of extractives) were obtained by applying cluster analysis to the three main components.
- Clusters 1, 2, 4, and 5 comprise only the reference and thermally modified samples at 180 °C (except SC200 and MB200), whereas cluster 1 comprises only SC20. Cluster 3 consists of 12 samples, mostly thermally modified at 200 °C and 220 °C, plus TE20 and TE180 samples.
- The yellowness (Y) of the wood has a very high dependence (r = 0.972) on the brightness (L*) of these samples.
- The coefficient of determination (r2 = 0.973) indicates a very high degree of explanation of the variable yellowness (Y) using the parameter L*, and also b*.
- A certain dependence (r = 0.714) is also noted between the yellowness (Y) and the yellowness index of extractives soluble in acetone Yi(Ac), whose relationship is described by the equation Y = −0.0951 × Y(Ac) + 23.3485.

Due to thermal modification above 200 °C, tropical wood loses its specific color, which makes individual species unique and interesting in terms of design. Although the color becomes homogeneous, the wood significantly loses its brightness and yellowness, and from temperatures of 220 °C, there are almost no differences in their color, as confirmed by the cluster analysis within the PCA. This may not always be desirable, because in some cases, consumers select a wood based solely on its decorative properties. Of course, the use of this type of wood will always depend on the specific application. And whether the color of the wood itself will be important at all, or whether other requirements from the advantage of ThermoWood itself will be prioritized.

The next phase of this research aims to focus more closely on the characteristics of preserved extracted substances from selected tropical wood species thermally modified at different temperatures using gas chromatography (GC-MS) and infrared spectroscopy (FTIR).

## Figures and Tables

**Table 1:** Characteristics of the analyzed wood species.

Trade Name (Samples Marking)	Latin Name	Occurrence	Color/Appearance
Spanish cedar (SC)	*Cedrela odorata*	Central and South America and the Caribbean; also, plantations	heartwood is light pink to reddish brown
Iroko (IR)	*Chlorophora excelsa*	Africa, especially in the Ivory Coast	heartwood is yellow to golden or medium brown, with color tending to darken over time; pale yellow sapwood is demarcated from the heartwood
Padouk (PA)	*Pterocarpus soyauxii*	Central and West Africa (e.g., Congo, Angola)	fresh heartwood is blood-red in color, changing to a dark purplish-brown with a red tinge over time
Meranti (ME)	*Shorea* spp.	Southeast Asia (e.g., Laos, Philippines)	core varies depending on the origin and species, ranging from brownish-pink to red to dark red; it darkens further in the light or, on the contrary, pales
Merbau (MB)	*Intsia* spp.	Southeast Asia (e.g., Burma, Indonesia), East Africa, and Australia (New Guinea)	fresh cut orangish-brown color, which ages to a darker reddish-brown; small yellow mineral deposits that can cause staining
Teak (TE)	*Tectona grandis*	Native to southern Asia; widely on plantations throughout tropical regions of Africa, Asia, and Latin America	heartwood tends to be a golden or medium brown, with color darkening with age; often with dark brown or black stripes (2–8 mm wide)

**Table 2:** Measured (L*, a*, b*, ISO brightness, and yellowness) and calculated (ΔE*) color values for samples of selected species of thermally modified tropical wood.

Wood Sample	Modification Temperature (°C)	L*	a*	b*	ΔE*	ISO Brightness	Yellowness (Y)
Sp. cedar (SC)	20 (ref.)	66.32 ± 0.34	12.24 ± 0.14	24.10 ± 0.14	-	20.06 ± 0.19	34.89 ± 0.24
	180	40.42 ± 0.68	14.67 ± 0.25	23.78 ± 0.46	26.02	7.99 ± 0.16	14.95 ± 0.27
	200	35.49 ± 0.79	13.33 ± 0.09	20.49 ± 0.14	31.06	6.74 ± 0.15	11.82 ± 0.24
	220	26.00 ± 0.60	8.30 ± 0.29	13.93 ± 0.47	41.77	5.10 ± 0.04	7.58 ± 0.14
Iroko (IR)	20 (ref.)	52.58 ± 1.26	9.81 ± 0.14	24.93 ± 1.23	-	10.99 ± 0.13	22.64 ± 0.29
	180	52.27 ± 0.61	10.86 ± 0.25	28.69 ± 0.30	3.92	11.88 ± 0.14	23.62 ± 0.26
	200	29.44 ± 0.79	10.58 ± 0.41	14.86 ± 1.16	25.25	5.67 ± 0.04	8.33 ± 0.25
	220	17.52 ± 1.07	2.91 ± 0.36	4.19 ± 0.50	41.32	4.63 ± 0.14	5.03 ± 0.13
Padouk (PA)	20 (ref.)	39.45 ± 0.81	29.00 ± 0.73	30.57 ± 1.35	-	5.32 ± 0.13	10.72 ± 0.37
	180	28.89 ± 0.87	26.53 ± 0.40	26.19 ± 0.62	11.70	4.52 ± 0.03	8.99 ± 0.12
	200	22.03 ± 0.41	9.44 ± 0.42	10.68 ± 0.74	32.90	4.72 ± 0.17	6.08 ± 0.23
	220	22.40 ± 0.51	3.92 ± 0.30	4.17 ± 0.28	40.21	5.51 ± 0.06	6.08 ± 0.09
Meranti (MT)	20 (ref.)	58.96 ± 0.31	10.09 ± 0.21	17.91 ± 0.24	-	18.66 ± 0.30	28.24 ± 0.40
	180	40.25 ± 1.17	9.26 ± 0.49	19.91 ± 1.06	18.83	8.64 ± 0.22	14.54 ± 0.58
	200	30.87 ± 1.12	10.57 ± 0.42	19.68 ± 0.84	28.15	5.75 ± 0.14	10.01 ± 0.36
	220	18.09 ± 1.11	6.13 ± 0.72	10.67 ± 1.79	41.70	4.27 ± 0.04	5.70 ± 0.11
Merbau (MB)	20 (ref.)	39.29 ± 3.26	13.54 ± 0.91	23.50 ± 4.55	-	7.27 ± 0.11	15.19 ± 0.21
	180	24.13 ± 0.29	11.70 ± 0.33	12.11 ± 0.39	19.06	5.40 ± 0.05	7.42 ± 0.09
	200	17.43 ± 0.91	4.24 ± 0.51	5.24 ± 0.13	29.97	4.65 ± 0.03	5.24 ± 0.05
	220	22.52 ± 0.42	6.06 ± 0.31	7.94 ± 0.54	24.07	5.22 ± 0.06	6.54 ± 0.05
Teak (TE)	20 (ref.)	40.38 ± 1.17	14.77 ± 0.23	30.19 ± 0.39	-	6.19 ± 0.09	13.62 ± 0.21
	180	33.18 ± 1.08	11.32 ± 0.22	21.05 ± 0.15	12.14	6.26 ± 0.08	11.82 ± 0.16
	200	19.49 ± 0.92	8.87 ± 0.54	10.27 ± 0.69	29.47	4.33 ± 0.06	5.56 ± 0.13
	220	15.05 ± 0.44	4.17 ± 0.31	5.21 ± 0.41	37.13	4.03 ± 0.12	4.47 ± 0.11

**Table 3:** Determined mass yields (w) and color characteristics (ΔE*, Yi) for extractives in selected solvents obtained from investigated thermally modified tropical wood species.

Wood Sample	Modification Temperature (°C)	Extractives in Acetone			Extractives in Ethanol			Extractives in Ethanol-Toluene		
		w (wt.%)	ΔE*	Yi	w (wt.%)	ΔE*	Yi	w (wt.%)	ΔE*	Yi
Sp. cedar (SC)	20 (ref.)	5.00 ± 0.00	-	40.70	5.13 ± 1.32	-	180.80	2.31 ± 0.60	-	49.40
	180	1.82 ± 0.13	15.81	62.40	2.95 ± 0.95	60.43	74.70	2.60 ± 0.24	33.85	97.00
	200	2.14 ± 0.06	29.73	85.00	4.37 ± 0.20	51.91	82.80	2.89 ± 0.65	30.99	97.40
	220	0.11 ± 0.00	49.90	107.20	3.30 ± 0.47	24.66	177.00	1.38 ± 0.04	76.42	164.90
Iroko (IR)	20 (ref.)	4.98 ± 0.00	-	28.90	2.44 ± 0.61	-	129.30	3.64 ± 0.82	-	103.40
	180	0.42 ± 0.04	9.93	35.90	1.40 ± 0.23	28.33	89.90	0.35 ± 0.05	36.00	149.60
	200	1.36 ± 0.11	95.50	89.50	3.59 ± 0.00	12.93	137.00	1.27 ± 0.16	24.90	135.10
	220	0.79 ± 0.06	72.33	137.70	2.26 ± 0.08	28.06	189.00	1.27 ± 0.01	63.78	228.10
Padouk (PA)	20 (ref.)	13.20 ± 0.00	-	210.80	4.81 ± 0.17	-	232.60	4.90 ± 0.00	-	271.70
	180	2.45 ± 0.36	16.53	199.40	5.05 ± 0.22	34.40	214.60	3.05 ± 0.03	10.53	251.80
	200	1.20 ± 0.08	35.96	215.70	0.70 ± 0.02	47.92	138.90	2.29 ± 0.26	10.68	242.30
	220	3.05 ± 0.08	28.04	159.00	1.69 ± 0.08	48.18	155.30	1.39 ± 0.07	38.87	189.00
Meranti (MT)	20 (ref.)	4.15 ± 0.00	-	15.00	1.23 ± 0.41	-	29.80	3.27 ± 0.40	-	17.80
	180	0.65 ± 0.08	38.02	54.10	3.57 ± 0.37	60.63	124.20	1.63 ± 0.37	56.62	111.60
	200	1.46 ± 0.12	83.58	125.00	1.57 ± 0.37	79.67	151.40	1.21 ± 0.06	62.49	105.40
	220	1.80 ± 0.02	66.87	112.90	1.70 ± 0.12	86.25	159.90	1.16 ± 0.17	64.50	111.20
Merbau (MB)	20 (ref.)	7.37 ± 0.57	-	116.70	4.86 ± 0.15	-	221.50	7.49 ± 0.02	-	202.80
	180	0.90 ± 0.03	26.44	99.00	4.56 ± 0.04	16.25	240.30	1.57 ± 0.22	10.71	209.60
	200	0.84 ± 0.03	31.94	169.40	4.21 ± 0.09	78.93	277.30	1.40 ± 0.05	15.07	169.80
	220	2.17 ± 0.13	47.37	189.10	1.69 ± 0.38	59.25	91.90	1.75 ± 0.23	36.04	134.20
Teak (TE)	20 (ref.)	4.6 ± 0.03	-	182.80	1.95 ± 0.09	-	153.90	1.27 ± 0.11	-	202.70
	180	2.57 ± 0.03	35.88	132.50	3.06 ± 0.18	27.61	125.20	1.38 ± 0.11	38.83	132.60
	200	1.75 ± 0.46	25.50	134.70	0.60 ± 0.03	35.93	85.40	1.33 ± 0.05	30.35	175.00
	220	0.83 ± 0.08	26.50	149.50	1.83 ± 0.01	22.49	163.70	3.05 ± 0.47	40.77	166.00

**Table 4:** The correlation coefficient of the wood yellowness variable (Y) concerning other parameters.

Variable	Y_i_(Ac)	Y_i_(Et)	Y_i_(Et-To)
Y (Yellowness)	−0.7076	−0.3219	−0.6022
	L*	a*	b*
	0.9719	0.2084	0.6141
	L*(Ac)	a*(Ac)	b*(Ac)
	0.6502	−0.5561	−0.7579
	L*(Et)	a*(Et)	b*(Et)
	0.2217	−0.2174	−0.2881
	L*(Et-To)	a*(Et-To)	b*(Et-To)
	0.5746	−0.5467	−0.6007

**Table 5:** Regression analysis for the dependent variable wood yellowness (Y).

N = 24	Regression Summary for Dependent Variable: Y R = 0.9866, R2 = 0.9733, Adjusted R2 = 0.9708 F (2.21) = 383.06, *p* < 0.0000, Std. The Error of Estimate: 1.3641			
	b*	b	t(21)	*p*-Value
Intercept		−5.7726 ± 0.7337	−7.8675	0.0000
L*	1.1630 ± 0.0537	0.6633 ± 0.0306	21.6611	0.0000
b*	−0.2555 ± 0.0537	−0.2388 ± 0.0502	−4.7595	0.0001
**N = 24**	**Regression summary for dependent variable: Y** **R = 0.7141, R2 = 0.5099, adjusted R2 = 0.4364** **F (3.20) = 6.9361, *p* < 0.0022, Std. The error of estimate: 5.9908**			
Intercept		23.7871 ± 3.6857	6.4540	0.0000
Yi(Ac)	−0.6029 ± 0.2488	−0.0810 ± 0.03343	−2.4238	0.0250
Yi(Et)	0.0597 ± 0.1978	0.0080 ± 0.0264	0.3022	0.7656
Yi(Et-To)	−0.1702 ± 0.2786	−0.0214 ± 0.0351	−0.6109	0.5481

## Data Availability

Data are available from the authors on request.
